# Vector-borne Infections

**DOI:** 10.3201/eid1705.110310

**Published:** 2011-05

**Authors:** Ronald Rosenberg, C. Ben Beard

**Affiliations:** Author affiliation: Centers for Disease Control and Prevention, Fort Collins, Colorado, USA

**Keywords:** Vector-borne infections, emerging diseases, arthropod vectors, bacteria, viruses, rickettsia, protozoa, helminths, parasites, perspective

## Abstract

Infections with vector-borne pathogens are a major source of emerging diseases. The ability of vectors to bridge spatial and ecologic gaps between animals and humans increases opportunities for emergence. Small adaptations of a pathogen to a vector can have profound effects on the rate of transmission to humans.

This issue of Emerging Infectious Diseases highlights the role of arthropod vectors in the origin and dissemination of emerging pathogens. As Woolhouse and Gaunt have pointed out (*1*), a substantial proportion of human pathogens are zoonotic and vector-borne, and they infect a substantial proportion of the world’s population. Vector-borne pathogens also are prominent contributors to emerging disease. There are 3 principal reasons for this influence.

First, most major classes of pathogens have evolved agents that are capable of being transmitted by blood-feeding arthropods: viruses (e.g., yellow fever virus, Rift Valley fever virus), rickettsiae (*Rickettsia rickettsii*, *R. typhi*), bacteria (*Borrelia burgdorferi*, *Francisella tularensis*), protozoa (genera *Plasmodium* and *Leishmania*), and helminths (*Onchocerca volvulus*, *Wuchereria bancrofti*). Fungi seem to be the only category not represented.

Second, vectors bridge barriers that would prevent transmission by direct contact among humans and especially between animals and humans. These barriers are not only spatial but behavioral and ecological. Transmission of yellow fever virus between arboreal monkeys and humans by mosquitoes is the classic example, but there are many others; transmission of *B. burgdorferi*, the agent of Lyme disease, between evasive forest rodents and humans by ticks is just as exemplary. In such cases, direct contact between feral host and human would rarely take place. A corollary of this ability to bridge environments occurs when animals or humans move the pathogen from one vector-capable region to another. The introduction of West Nile virus into the United States in 1999 was a dramatic example, as was the recent introduction of Usutu virus to Europe from Africa in migrating birds (*2*). The potential for vector-borne zoonotic transmission to adapt to vector-borne human-to-human transmission is exemplified historically by dengue virus and *Plasmodium* spp., and more recently by Zika virus (*3*) and probably *P. knowlesi* (*4*).

Third, the complexity of vector transmission offers the pathogen increased opportunities to evolve. In almost no instances is the arthropod simply a vessel for transmission. Usually, the pathogen must move from the gut to the feeding apparatus to be transmitted. Mechanisms range from the relatively simple, as with the plague bacillus, *Yersinia pestis*, to the elaborately intricate, as with parasites in the genera *Plasmodium* and *Leishmania*. In these examples, the pathogen replicates in some fashion, which makes it dependent on an invertebrate host physiology much different from what it will encounter in its various vertebrate hosts. As a consequence, epidemic emergence can result from enhanced transmission independent of increased pathogenicity to humans. This is especially true of the arthropod-borne viruses (arboviruses) that infect humans, all of which are RNA viruses and have high potential mutability. A notable recent example is the chikungunya virus epidemic that swept through the Indian Ocean region beginning in 2006 and which is believed to have infected >2 million persons. A single-nucleotide polymorphism (SNP) in the virus genome accelerated its replication in the relatively common mosquito *Aedes albopictus*, usually a poorer host than *Ae*. *aegypti* mosquitoes (*5*). There is also evidence that an SNP enabled Venezuelan equine encephalitis virus to jump vectors, sparking the 1993 epidemic in Mexico (*6*), and it might have been an SNP in West Nile virus that increased its virulence to birds and influenced the shape of the epidemic in the United States (*7*). In none of these examples was increased pathogenicity to humans an apparent seminal factor in the epidemics.

Complexity of epidemiology and adaptive plasticity of pathogen and arthropod make the vector-borne diseases especially difficult to control, much less to eradicate. Vaccines are unavailable for all but a few diseases; and even when they are available, as for yellow fever, prevention can be difficult to achieve. The yellow fever epidemic that began in Uganda at the end of 2010 was the first in that country in 20 years. Tools for treatment are nearly as scarce. Falling behind in the race to keep up with developing resistance of *P. falciparum* to artemisinins is a specter that haunts malariologists, and treatment for visceral leishmaniasis remains too expensive and complicated to be widely practiced where it is most needed.

The constant development of pesticide resistance is even more worrisome than drug resistance because a pesticide can often be used to suppress vectors of many different pathogens. Even when pesticides are efficacious, their effectiveness is often compromised by human behavior and vector biology, as is often seen in campaigns against dengue. Changes in climate, land use, and transport will affect rates of pathogen emergence in ways we poorly understand. Fortunately, there is a growing appreciation by scientists and by funding agencies (*8*) that characterizing factors that influence pathogen and disease emergence are worthy goals for investigation, especially in those tropical environments where rapid change is most likely to incubate new pathogens.
